# Mushroom Polysaccharides: Chemistry and Antiobesity, Antidiabetes, Anticancer, and Antibiotic Properties in Cells, Rodents, and Humans

**DOI:** 10.3390/foods5040080

**Published:** 2016-11-29

**Authors:** Mendel Friedman

**Affiliations:** Western Regional Research Center, Agricultural Research Service, U.S. Department of Agriculture, 800 Buchanan Street, Albany, CA 94710, USA; mendel.friedman@ars.usda.gov; Tel.: +1-510-559-5615

**Keywords:** bioactivity, biomarkers, polysaccharides, mushrooms, chemistry, antibiotics, obesity, diabetes, cancer, health-promoting food additives, functional food, research needs

## Abstract

More than 2000 species of edible and/or medicinal mushrooms have been identified to date, many of which are widely consumed, stimulating much research on their health-promoting properties. These properties are associated with bioactive compounds produced by the mushrooms, including polysaccharides. Although β-glucans (homopolysaccharides) are believed to be the major bioactive polysaccharides of mushrooms, other types of mushroom polysaccharides (heteropolysaccharides) also possess biological properties. Here we survey the chemistry of such health-promoting polysaccharides and their reported antiobesity and antidiabetic properties as well as selected anticarcinogenic, antimicrobial, and antiviral effects that demonstrate their multiple health-promoting potential. The associated antioxidative, anti-inflammatory, and immunomodulating activities in fat cells, rodents, and humans are also discussed. The mechanisms of action involve the gut microbiota, meaning the polysaccharides act as prebiotics in the digestive system. Also covered here are the nutritional, functional food, clinical, and epidemiological studies designed to assess the health-promoting properties of polysaccharides, individually and as blended mixtures, against obesity, diabetes, cancer, and infectious diseases, and suggestions for further research. The collated information and suggested research needs might guide further studies needed for a better understanding of the health-promoting properties of mushroom polysaccharides and enhance their use to help prevent and treat human chronic diseases.

## 1. Introduction

**Figure 1:**
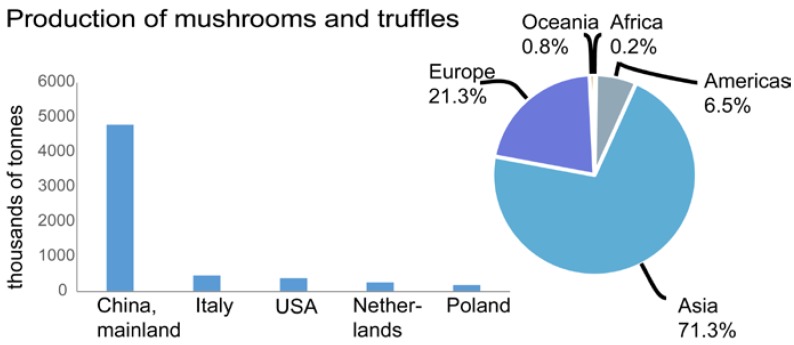
Worldwide production of mushrooms and truffles, average from 2004 to 2014 [5].

**Figure 2:**
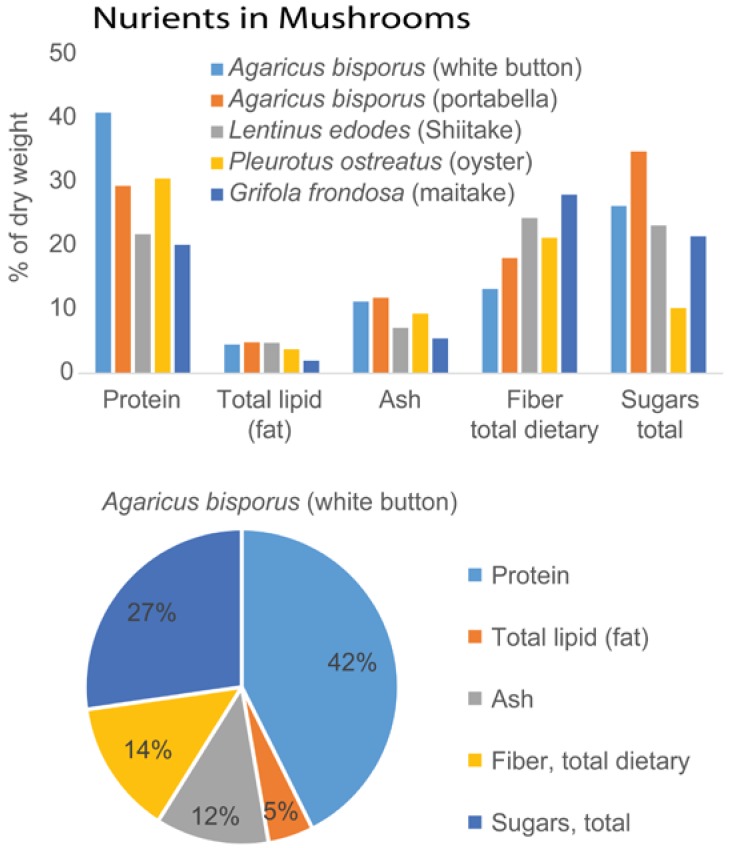
Nutrient content of five select mushrooms as reported by the National Nutrient Database for Standard Reference, Release 28 [6].

Mushrooms are a widely consumed, low-calorie, low-cholesterol, and low-sodium health-promoting food. Some of these health-promoting properties have been attributed to the polysaccharides produced by different varieties mushrooms, as discussed in this review. Figure 1 shows that China dominates over other countries in the global production of mushrooms and Figure 2 shows that the protein, fat, ash (mineral), sugar, and fiber content varies widely among five widely consumed edible mushrooms.

**Figure 3:**
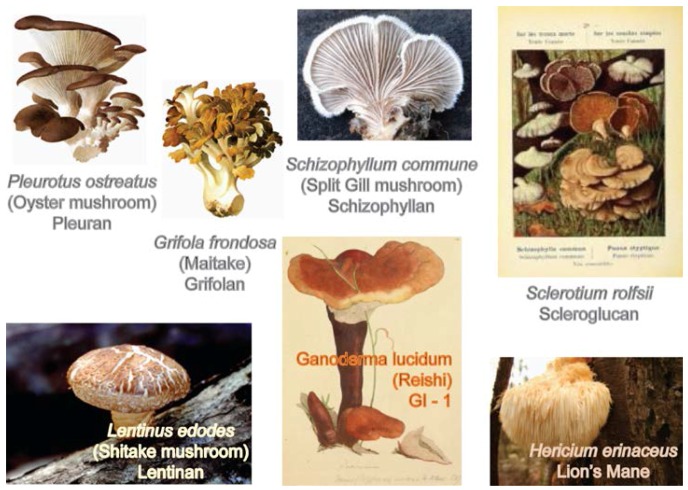
Mushrooms containing β-(1→3)-glucans with β-(1→6) branching).

**Figure 4:**
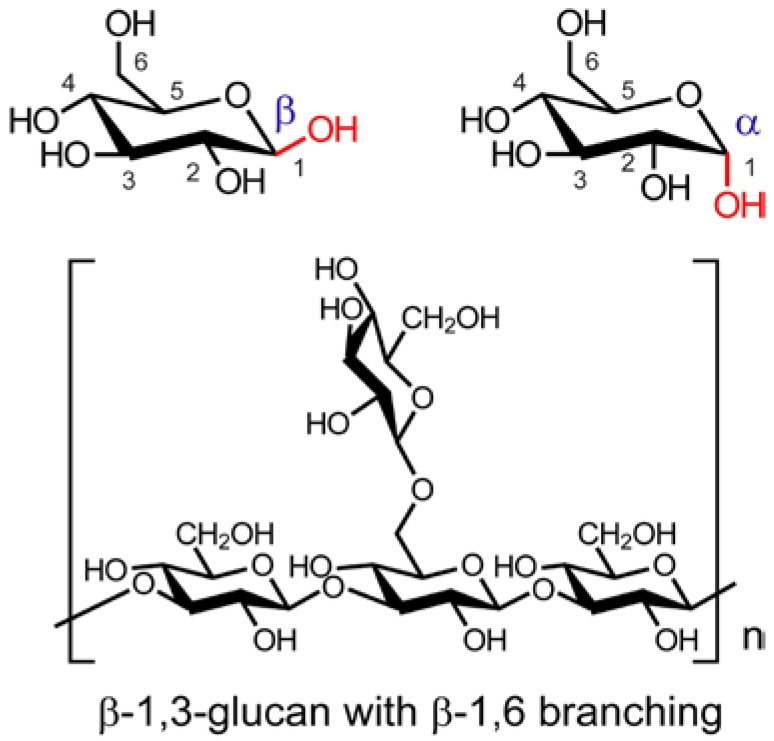
Basic structure of a typical mushroom polysaccharide. Variations include 1→4 linkages, α-glucan moieties, and alternate sugars.

**Figure 5:**
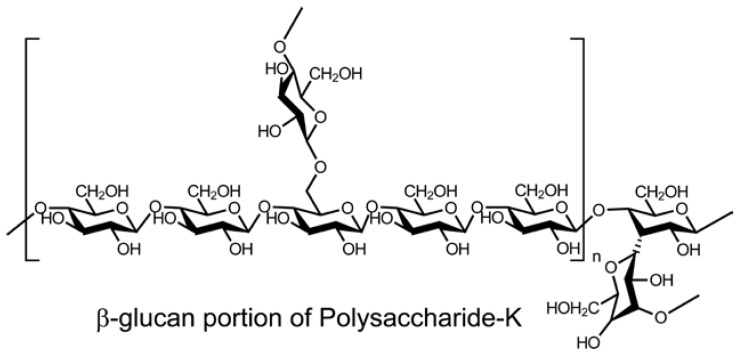
β-Glucan/protein complex isolated from *Trametes versicolor* (Turkey Tail mushroom) containing 25%–38% protein. 94 kDa macromolecule. β-1→4-glucan backbone with 1→3/1→6 branching β-glucan. The protein consists of 28% acidic amino acids [7,8,9].

Aida, et al. [1] reviewed the prospect of mushrooms as a source of prebiotics that have the potential to promote human health; prebiotics are defined as selectively fermented food ingredients that induce changes in the composition and activity of the gastrointestinal tract microbiota that confer nutritional and health benefits to the host. Such compounds include mushroom polysaccharides, most of which are β-glucan polymers, with the main chain consisting of β-(1→3) linkages with some β-(1→6) branches as well as chitin, mannans, galactans, and xylans. Digestive enzymes secreted by the pancreas cannot hydrolyze the β-glucosidic bond, which means the non-digestible mushroom carbohydrates can act as prebiotics in the digestive tract. Non-digestible oligosaccharides consist of two to three monosaccharides without nutritional value. For prebiotics from foods to have their beneficial effects they should not be structurally changed by food-processing conditions, such as heat and microwaves. Mushrooms and mushroom-derived polysaccharides, illustrated in Figure 3, Figure 4 and Figure 5, have been shown to have therapeutic properties against metabolic syndrome, which is characterized by obesity, hyperglycemia associated with diabetes, hypercholesterolemia, and hypertension [2,3,4].

Obesity is a widespread problem; indeed, in the United States it is estimated that more than one third of adults (about 80 million individuals) are obese [10,11]. There also seems to be substantial educational disparity in obesity among United State adults; those with higher education are on average less obese. As, reviewed elsewhere [12,13], obesity and being overweight are consequences of excessive adipogenesis. Fat molecules (triglycerides) consist of three molecules of fatty acids linked as esters to glycerol that coalesce into droplets in the cytosol, accounting for most of the volume of fat cells, called adipocytes, that are the cells specialized for fat storage in adipose tissue. Adipogenesis—the generation of adipocytes—is regulated by adipogenic transcription factors, including peroxisome proliferator activated receptor-γ (PPARγ), sterol regulatory element-binding protein 1c (SREBP-1c), and the CCAAT (cytosine-cytosine-adenosine-adenosine-thymidine)-enhancer binding protein (C/EBP) family. It seems that PPARγ is induced during adipogenesis and is necessary for this process. Adipogenesis and insulin sensitivity in obesity are also reported to be regulated by retinoid-related orphan receptor γ (RORγ) through its target gene MMP3 (matrix metalloproteinase-3 gene), which controls adipocyte size and insulin resistance in obesity. In addition, chemerin (an adipocyte-secreted protein), the angiotensin system, and estrogen receptors are reported to be involved in adipogenesis in vivo. Each of these factors has the potential to serve as a target that might protect against obesity and related metabolic syndromes such as overlapping obesity-associated insulin resistance (diabetes).

**Figure 6:**
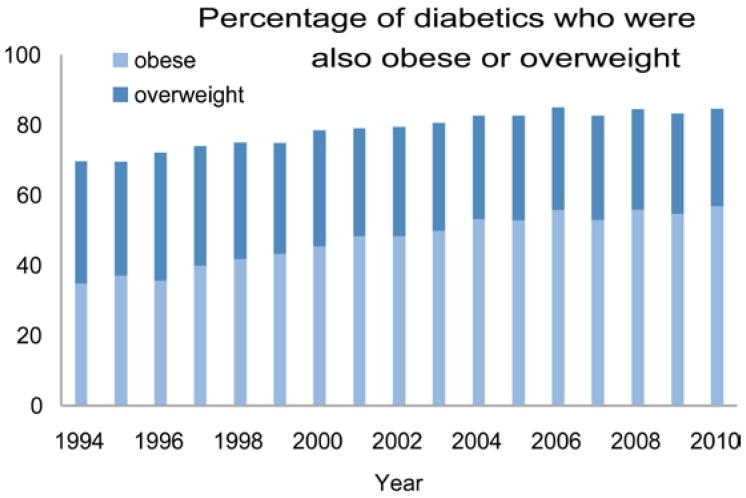
Age-adjusted percentage of US adults aged 18 years or older with diagnosed diabetes who were obese or overweight. Adapted from United States Centers for Disease Control, Diabetes Public Health Resource Statistics [16].

Diabetes is a disease characterized by hyperglycemia resulting from defects in insulin metabolism. Insulin is a hormone secreted by β-cells in pancreatic islets that primarily stimulates cells to take up glucose, although it also affects metabolism of all carbohydrates, fats, and protein. Type 2 diabetes (TD2) is caused by acquired resistance to insulin-stimulated glucose uptake by cells combined with inadequate compensatory insulin production to overcome the resistance. Type 1 diabetes (TD1) is caused by insufficient insulin production by the pancreas β-cells [14,15]. As shown in Figure 6, TD2 is related to obesity and may be substantially prevented or managed by changes in lifestyle.

The lack of insulin leads to abnormalities in the metabolism of essential nutrients, including carbohydrates, lipids, and proteins. Data from the American Diabetes Association [17] on the prevalence of diabetes show that in 2012, 29.1 million American, or 9.3% of the population, had TD2 and about one million, TD1. The worldwide incidence of type 2 diabetes is expected to increase to 300 million individuals by the year 2025 [18]. Socioeconomic factors seem to be responsible for reported differences in the incidence among ethnic/racial groups [19].

To help overcome the so-called metabolic syndrome that includes obesity and diabetes using dietary modifications, this paper: (a) describes methods used to isolate, characterize, and analyze polysaccharides from selected mushroom cultivars; (b) outlines approaches used to measure antiobesity, antidiabetic, anticancer, and antibiotic properties of mushroom polysaccharides and selected mushroom cultivars in cells, rodents, and humans; (c) describes mechanisms involving microbiota and immunostimulatory and other biomarkers and biological signalling pathways that might govern these properties; and (d) suggests research needs to further enhance the health benefits of mushroom polysaccharides. Unless otherwise indicated, the mentioned suggested significance of the results is that of the authors of the cited papers.

## 2. Isolation and Characterization of Mushroom Polysaccharides

Mushroom β-glucan polysaccharides are fibers that have numerous reported biological functions [20]. The submerged cultivation of mushrooms seems to be a promising, and possibly more efficient, alternative method to that of conventionally grown mushrooms for the production of mushroom mycelia and bioactive metabolites, including polysaccharides [21]. Polysaccharides from *Hericium erinaceus*, known as the Lion’s Mane mushroom, have been of particular interest because of the bioactivities that have been attributed to them [22]. Here, we offer capsule summaries of the chemical and analytical methods that have been use to isolate and characterize polysaccharides from selected mushroom cultivars.

Wu, et al. [23] used a range of methods—colorimetry with iodine and KI, high performance size exclusion chromatography coupled with multi angle laser light scattering and refractive index detection (HPSEC-MALLS-rid analysis), gas chromatography-mass spectrometry (GC-MS) analysis, and saccharide mapping based on carbohydrate gel electrophoresis (PACE)—to identify polysaccharides isolated from *H. erinaceus* fruiting bodies collected from different parts of China.

The results from the different techniques show similarity in their profiles, including molecular weights, the composition of monosaccharides, and the glycosidic linkages in the polysaccharides, suggesting that the described experimental approach could be used for the quality control of polysaccharides in edible and medicinal mushrooms, as well as in commercial mushroom products.

Related studies by several investigators include the following multifaceted observations: (a) During growth and maturation of the fruiting bodies, total polysaccharide levels increased and protein content decreased [24]; (b) Polysaccharides collected from fruiting bodies of different regions of China had similar molecular weights, monosaccharide compositions, and glycosidic linkages [23]; (c) Polysaccharides from eight species exhibited strong antioxidant activity in the DPPH radical scavenging assay as well as strong inhibitory properties in the proliferation of tumor cells [25]; (d) A bismuth-polysaccharide complex inhibited the growth of *Helicobacter pylori* bacteria reported to contribute to the cause of human ulcers [26]; (e) *H. erinaceus* and other mushroom varieties are reported to be a useful source of lignocellulose-degrading (ligninolytic) carboxymethyl cellulase and laccase enzymes, suggesting that mushrooms can provide enzymes for use in the development of bioenergy [27].

Using high-performance size exclusion chromatography (HPSEC) to establish the degree of homogeneity and molecular weights, Malinowska, et al. [28] evaluated several factors that govern mycelial growth in a submerged culture of *H. erinaceus* and production of intracellular polysaccharides (IPS) and exopolysaccharides (EPS). They found that the kinetics of EPS biosynthesis in a bioreactor proceeded until depletion of the carbon source in the medium on day 14 of cultivation, suggesting the value of the method for large-scale production of bioactive polysaccharides from *H. erinaceus*.

Zhu, et al. [29] describe the use of an enzyme-mixture (cellulase, pectinase, and tryptinase) assisted extraction of polysaccharides from the fruiting bodies of *H. erinaceus* mushrooms using response surface methodology and the Box-Behnken design based on single-factor and orthogonal experiments to optimize the extraction conditions (pH 5.7, 52.0 °C, and 33.8 min). The optimal extraction produces a high yield (13.5%) corresponding to an increase of 67.7% compared to hot water extraction. The functional groups of the two extracted polysaccharides seemed to be identical but differed in their conformations. The authors suggest that the method might be useful for extracting polysaccharides from other mushroom species.

The repeating basic units of β-glucan polysaccharides derived from edible shiitake (*Lentinus edodes*) mushrooms consist of five linearly linked (1→3)-β-glucose residues and two 1-6-β-glucopyranoside branches [30]. To determine the β-glucan content of shiitake mushrooms, these authors used a mushroom specific β-glucan kit on different sections of fruiting bodies and mycelia of ten shiitake mushroom cultivars. This involved hydrolysis of the shiitake samples with 37% HCl for 45 min at 30 °C followed by workup of the reaction mixture. The β-glucan content was determined by subtracting the α-glucan value, determined by an enzymatic method, from the total value. The measured β-glucan values ranged (in %) from 20.06 to 44.21 in the pileus sections and from 29.74 to 56.47 in the stipe section, showing that β-glucan content varies significantly among cultivars and is higher in the stipe of the fruiting bodies than in the pileus. The authors mention related analyses by other investigators. The detailed description of analytical methods used in this study for the shiitake mushrooms should be applicable to other cultivars. See also Kim, et al. [31] for a detailed description of the isolation and determination of the composition of a polysaccharide from a bioprocessed liquid culture of *Lentinus edodes* mushrooms that might be helpful in studies with other mushroom cultivars.

Silveira, et al. [32] describe the chemical structure of an exopolysaccharide isolated from *Pleurotus sajor-caju* mushrooms. After extraction, the polysaccharide was purified by freeze-thawing and dialysis and its structure was then characterized by GC/MS and NMR spectroscopy to show that the polymer was a mannogalactan with a minimum chain length of (1→6)-linked α-d-Galp and 3-*O*-methyl-α-d Galp units composed of mannose (37.0%), galactose (39.7%), and *O*-methyl-galactose (23.3%).

In an investigation of the relationship between structure and the reported antihyperglycemic effect of polysaccharides from *Catathelasma ventricosum* mushrooms, Liu et al. [18,33,34], describe their isolation and characterization. The freeze-dried powdered mushrooms (200 g) were defatted and decolorized with 95% ethanol and the suspension was centrifuged. The supernatant was discarded and the residue was extracted thrice with distilled water (4 L, 85 °C, 3 h). The combined extracts were concentrated in a rotary evaporator. After removal of protein, the extract was dialyzed in a DEAE cellulose dialysis bag against distilled water for 2 days to remove low molecular weight compounds. The high-molecular weight polysaccharide that was retained in the dialysis bag was further purified by the addition of 3-fold volumes of ethanol (48 h, 4 °C) and collection of the precipitate by centrifugation (5000 rpm, 10 min). The precipitate was then lyophilized to a mixture of polysaccharides (32.77 g). The resulting powder contained more than 99% polysaccharides, was negative in the Bradford test for proteins, and showed no absorption at 280 and 260 nm in the UV spectrum, indicating the absence of proteins and nucleic acids. Analysis by Fourier transform infrared spectroscopy (FT-IR) and other analytical methods revealed the presence of six main peaks with average molecular weights ranging from 3.7 × 10^3^ to 1.7 × 10^7^ Da using dextran-T as a standard. A hydrolyzed and acetylated sample was then subjected to GC analysis that showed the presence of glucose with an α-d-glucopyranose conformation (93.5%), fucose (0.7%), mannose (1.4%), and galactose (4.3%). The described methodology should be useful for the structural elucidation of polysaccharides from other mushroom cultivars.

The vast literature (more than 1600 citations in the PubMed) on the isolation, structural elucidation, and bioactivities of mushroom polysaccharides includes reports on the following additional mushroom cultivars: *Agaricus blazei* [35], *Agaricus brasiliensis* [36], *Amanita ponderosa* [37], oyster mushroom [38], *Auricularia polytricha* [39], *Boletus edulis* [40], *Cookeina tricholoma* [41], *Cordyceps militaris* [42], *Entoloma lividoalbum* [43], *Gleoestereum incarnatum* [44], *Ganoderma lucidum* [45], *Grifola frondosa* [46,47], *Hohenbuehelia serotina* [48], *Hypsizygus marmoreus* [49], *Iliodiction cibarium* [50,51], *Lactarius deliciosus* [52], *Lentinus edodes* [31,53], *Macrolepiota dolichaula* [54], *Phellinus igniarius* [55], *Phellinus linteus* [56], *Phellinus pini* [57], *Pholiota adiposa* [58], *Pholiota nameko* [59], *Pleurotus eryngii* [60], *Pleurotus ostreatus* [55,61], *Termitomyces heimii* [62], *Tricholoma matsutake* [63,64,65], *Tricholoma mongolicum* [66]. A journal reviewer noted that it is, however, important to determine if the isolated polysaccharides contain polyphenolic or other bioactive compounds that might affect their activities.

### 2.1. Enhanced Biosynthesis of Mushroom Polysaccharides

In principle, it might be possible to increase the polysaccharide content of mushrooms by amplifying the genes that govern their biosynthesis using molecular engineering techniques. The following recent studies describe successful approaches that show that this is indeed the case. (a) Chai, et al. [67] achieved the overproduction of β-glucans in *Pleurotus ostreatus* mushrooms by promoter engineering in which the promoter for the 1,3-β-glucan synthase gene was replaced by the promoter of glyceraldehyde-3-phosphate dehydrogenase gene of *Aspergillus nidulans*. The transformation produced 32% to 131% higher yield of β-glucans than the wild type; (b) Ji, et al. [68] improved polysaccharide production by engineering the biosynthetic pathway in *Ganoderma lucidum* through the overexpression of the homologous UDP glucose phosphoglucomutase gene. The intracellular and extracellular polysaccharides were higher by 42% and 36% than those of the wild type strain; (c) Meng, et al. [69] used the expression of the *Viteoscilla* hemoglobin gene to increase the production of both extracellular and intracellular polysaccharides in *Ganoderma lucidum* by 30.5% and 88.2%, respectively; and (d) UDP-glucose phosphorylase seems to be a key enzyme that is involved in polysaccharide biosynthesis [70], suggesting that amplification of the genes that govern the formation of enzymes involved in the biosynthesis of polysaccharides might induce the formation of high-polysaccharide mushrooms [71]. These pioneering studies demonstrate the potential of gene manipulation to enhance the production of polysaccharides.

### 2.2. Effect of γ-Radiation on Fractionation of Polysaccharides

Khan, et al. [72] examined the potential of γ-irradiation to increase the percentage of the soluble fraction as well as the effect on structural, functional, and antioxidant properties of the resulting smaller size molecules. The results show that irradiation with 50 kGy doses that are much higher than those used with mice mentioned below (4 to 8 Gy) induced a significant reduction in molecular weights due to bond cleavage, enhanced antioxidant activity, and increased functional properties associated with reduced viscosity, high solubility, and bile acid binding capacity, suggesting that γ-irradiation might facilitate the use of mushroom β-glucans in various food formulations.

## 3. Antioxidative, Immunostimulating, and Anti-Inflammatory Properties

The beneficial effect of antioxidants is due to their ability to destroy reactive oxygen species (ROS) that damage DNA and essential proteins. Antioxidative activities of polysaccharides in vivo are usually accompanied by increased activities in liver oxidative enzymes (catalase, glutathione peroxidase, superoxide dismutase) and increased glutathione and malondialdehyde levels [73,74]. The following selected studies show that antioxidative and concurrent immunostimulating properties of mushroom polysaccharides contribute to their bioactivities: (a) Antioxidative and immunostimulating properties of a polysaccharide isolated from *Cordiceps militaris* mushrooms seem to be responsible for the suppression of in vivo growth of melanoma in an mouse model [75,76]; (b) Antioxidative properties of a crude polysaccharide from *Inonotus oblique* mushrooms, widely used as a folk medicine in Russia, seem to contribute to the medicinal and nutritional properties of this mushroom [77]; (c) A polysaccharide from *Hericium erinaceus* mushrooms had strong in vitro antioxidant activity and protected mice against liver damage induced by carbon tetrachloride [78]; (d) A fucogalactan isolated from the edible mushroom *Macrolepiota dolichaula* exhibited antioxidant and immunostimulating (macrophage, splenocyte, and thymocyte activation) properties in vitro [54], (similar results were observes with a β-glucan isolated from the edible mushroom *Russula albonigra*) [79]; (e) A purified polysaccharide from *Pleurotus nebrodensis* improved immunity and coordinated innate immunity and inflammatory responses by activating macrophages [80,81]; (f) An exopolysaccharide from the medicinal mushroom *Clitocybe maxima* enhanced the immune response and inhibited tumor cells in mice [82]; (g) A polysaccharide from *Hericium erinaceus* exhibited both antioxidant and neuroprotective effects on Aβ-induced neurotoxicity in neurons [83]; (h) A polysaccharide from *Agaricus brasiliensis* induced immunostimulation in mice (increased spleen and thymus indexes) and increased RAW 264.7 cell proliferation in vitro [84]; (i) A related study found that the polysaccharide from this mushroom showed strong in vitro free radical scavenging activity [85]; (j) Polysaccharides extracted from the edible mushroom *Tricholoma mongolicum* exhibited in vitro antioxidant activities in a dose-dependent manner [66]; (k) Ultrasonically extracted *Ganoderma* β-d-glucans have higher molecular weights and optimal degree of branching, and better in vitro antioxidant activity in comparison with conventional extraction methods [86]; (l) A water-soluble β-glucan isolated from hot water extract of the fruit bodies of the edible mushroom *Entoloma lividoalbum* stimulated the production of macrophages, splenocytes, and thymocytes and exhibited hydroxyl and superoxide radical scavenging activities and reducing properties [43]; (m) A fucogalactomannan from *Tylopilus ballouii* mushroom inhibited superoxide and hydroxyl radicals with IC50 values of 1.25 and 1.6 mg/mL, respectively, and reduced edema by up to 56% in an anti-inflammatory assay [87].

The following additional immunomodulating observations deserve special mention. The polar fraction the β-glucan-rich mushroom preparation AndoSan™ that consists of a mixture of an extract of *Agaricus blazei* Murill (82.4%), *Hericium erinaceus* (14.7%), and *Grifola frondosa* (2.9%) significantly inhibited the activity of the tumor associated protease, legumain, in RAW 264.7 macrophage cells [88]. It seems that the immunomodulating polysaccharides (β-glucans) may contribute to the observed anti-allergic/-asthma anti-infection, and antitumor properties in mice, and anti-inflammatory effects in inflammatory bowel disease [89]. These beneficial effects seem to be mediated through the stimulation of innate immune cells, including monocytes, natural killer (NK) cells, and dendritic cells and to changes in the T-helper cell (T_h_1/T_h_2) ratio (balance) and inflammation. Surprisingly, administration of AndoSan™ to 40 patients with multiple myeloma scheduled to undergo high dose chemotherapy for seven weeks did not result in statistically significant differences in treatment response and overall survival [90]. It is also noteworthy that (a) an *Agaricus blazei* extract enhanced the immune response elicited by DNA vaccine against foot-and-mouth disease [91]; (b) *Hericium erinaceus* extracts protected mice against *Salmonella* Typhimurium mortality [92]; and (c) a polysaccharide isolated from liquid culture of *Lentinus edodes* mushroom mycelia containing black rice bran protected mice against both *Salmonella*-induced endotoxemia and salmonellosis through upregulation of the immune reaction [31,93].

Although mushrooms are a potential source of anti-inflammatory compounds [94,95,96], it is not possible to know for certain to what extent polysaccharides contribute to their biological functionalities. Moreover, only limited information seems to be available on the chemical basis of the numerous bioactivities of mushroom polysaccharides [6]. Although the biological activities of polysaccharides seem to be related to their structure or conformation properties [97], a journal reviewer noted that the abilities of polysaccharides to bind amino acids, peptides, proteins, polyphenols, steroids, etc. might also affect their biological activities. Nevertheless, the cited studies suggest the potential value of polysaccharides as antioxidative, immunostimulatory, and anti-inflammatory food additives.

## 4. Role of Gut Microbiota in Obesity and Diabetes

The beneficial impact of dietary mushroom polysaccharides on gut microbiota is currently an active area of research [10,98,99,100,101]. Here, we briefly mention the proposed impact of mushrooms and their polysaccharides on gut microbiota in relation to obesity and diabetes. The apparent association of obesity with the systemic immune inflammatory index [102] will not be covered here.

### 4.1. Definitions and Significance of Prebiotics, Probiotics, and Synbiotics

The following definitions and brief mention of their significance that are relevant to theme of this review have been adapted from the literature [103,104,105,106,107,108,109,110,111].

- Prebiotics are post-ingestion fermentable food ingredients such as non-digestible polysaccharides (β-glucans) and fibers that induce changes in the gastrointestinal microbiota that result in improved nutritional and health benefits to the host. Prebiotics of low-molecular-mass oligosaccharides are more rapidly and selectively fermented by *Bifidobacteria* and lactobacilli than higher molecular weight polysaccharides [110]. Prebiotics including polyphenol-rich apple and grape pomace polyphenols [112,113,114], can beneficially affect disorders of the digestive and immune systems, hypertension, appetite and obesity. Commercial prebiotics include the following non-digestible oligosaccharides: inulin, fructo-oligosaccharides (FOS), and galacto-oligosaccharides (GOS). The fermentation of non-digestible long-chain β-glucans from mushrooms to form short chain fatty acids (SCFAs) provides a potential source of new prebiotics [110]. Because *Ganoderma lucidum* reduces obesity in mice by modulating microbiota, Delzenne and Bindels [99] suggest that extracts of this mushroom might be considered a new prebiotic to treat obesity.
- Probiotics are preparations containing safe live gut microorganisms, usually *Bifidobacteria* and *Lactobacilli*, that are tolerant to the action of gastric and bile actions and which can alter the microflora of the digestive tract resulting in beneficial health effects to the host. Probiotics such as yogurt are reported to ameliorate adverse health syndromes (allergy, asthma, cancer, anxiety, and depression). Multifunctional prebiotics and probiotics have the potential to control obesity by helping regulate gut microbiota, food intake, and body weight. They therefore might be useful in the treatment of obesity, hyperglycemia, and dyslipidemia [115].
- Synbiotics are products containing both prebiotics and probiotics and refer specifically to the utilization of prebiotics by probiotic bacteria that form fermentation products that are more effective in ameliorating adverse health effects via modulation of the intestinal flora than are placebos [116].

### 4.2. Modulation of Gut Microbiota by Prebiotic Mushroom Polysaccharides

Obesity arises from the consumption of energy-rich foods that adversely affect the energy homeostasis of the host, whereby energy intake and energy expenditure are out of balance. Reducing energy density of the diet can help control obesity. In humans, weight loss induces changes in gut microflora. Dietary ingredients such as indigestible carbohydrates that are fermented in the gut to short-chain fatty acids can provide a new source of energy that helps restore the energy balance, thus ameliorating obesity [103]. Gut microbiota are also reported to affect obesity by helping to balance immunity and the nutritional status of the host [105]. The fat-lowering antiobesity potential of probiotics seems to be due to their ability to beneficially influence the ratio of two major classes of gut bacteria, *Bacteroides* and *Firmicutes*.

Murphy, et al. [111] suggest that the mechanism of the impact of high-fat diet (HFD) on gut microbiota is more complex than just the influence on energy balance. They showed that HFD-induced changes in gut microbiota leads to a decrease in *Bacteroides* and an increase in *Firmicute* bacteria, and the mechanism of this effect is associated with improved energy harvest and storage and enhanced gut inflammation and permeability. They concluded that a better understanding of the mechanism might facilitate the regulation of dysbiosis and the enhancement of antiobesity effects. The overall mechanism seems more complex in view of the observation that gut microbiota are also reported to reduce the expression of two genes coding for fat-suppressing neuropeptides that may have an adverse effect on body fat reduction [117].

Studies with other food groups also provide support for such a mechanism. Results from studies by Seo, et al. [118] on the alleviation of obesity in mice by fermented green tea extract seem to reinforce this mechanism. In addition to the antiobesity effects, the extract restored the beneficial gut microbiota composition (*Firmicutes/Bacteroides* and *Bacteroides*/*Prevotella* ratios), the balance of which, as mentioned, is related to HFD-induced development of obesity and insulin resistance. Related mechanistic aspects of changes in gut microbial population and are reported by Han, et al. [119]. These authors found that pyrosequencing of fecal microbiota and microarray analyses of blood samples showed that fresh and fermented kimchi interventions induced differential effects on the obesity-related clinical parameters. Consumption of ginseng also induced weight loss in obese Korean women that seems to be related to changes in the gut microbiota [120].

In relevant observations, Dutton and Turnbaugh [106], suggest that a metagenomic view of human nutrition ranging from the biotransformation of food to digestion and utilization, including microbial metabolism, might facilitate an exploitation of nutritional and health benefits of different food categories. Nieuwdorp, et al. [121] suggest that controlled studies involving perturbation of microbial communities in animal and human models may help identify therapeutic targets in the microbiota. Modulating microbiota with the use of antibiotics, prebiotics, and probiotics may also improve glucose metabolism and insulin resistance associated with type 2 diabetes; note that the mechanism that seems to govern the association of specific gut microbiota and metabolic disease include improved utilization dietary energy and changes in host-gene expression and gut permeability. Relevant studies by Huang et al. [122,123] show that polysaccharides from *Pleurotus tuber-regium* mushrooms exhibited antihyperglycemic properties and attenuated oxidative stress in diabetic rats on a high fat diet. Holmes [124], and Chang, et al. [98] showed that *Ganoderma lucidum* mushrooms reduce obesity in mice by modulating the composition of the gut microbiota. It is not known if polysaccharides contribute to this effect. These considerations suggest the need to better define the possible roles of gut microbiota in the polysaccharide-induced attenuation of obesity and hyperglycemia.

## 5. Antiobesity Effects

### 5.1. In Cells and Rodents

Obesity adversely affects immune, inflammatory, and platelet biomarkers [102]. Several studies have investigated the antiobesity effect of mushroom polysaccharides from different sources on either cells or rodents, and examples follow below.

#### 5.1.1. Coriolus Versicolor

Mao, et al. [47] found that polysaccharide from the medicinal mushroom *Coriolus versicolor* activated mouse splenocytes through the MAPK and NF-κB signaling pathways, suggesting binding to immunoregulators that induces an immunopotentiating effect.

#### 5.1.2. Tremella Fuciformis

Jeong, et al. [125] found that a polysaccharide from Hinmogi (*Tremella fuciformis*) edible mushrooms inhibited the differentiation of 3T3-L1 adipocytes by reducing the mRNA expression in the cells of PPARγ, C/EBPα, and the leptin hormone in a dose-dependent manner, suggesting the potential value of the polysaccharide as an antiobesity prebiotic.

#### 5.1.3. Ganoderma Lucidum

The treatment of adipocytes (fat cells) with the dietary supplement of *Ganoderma lucidum* mushrooms known as ReishiMax suppressed the expression of adipogenic transcription factors PPAR-γ, sterol regulatory binding element protein-1c (SREBBP-1c) and CCAAT/enhancer binding protein-α (C/EBP-α), as well as the following enzymes and proteins associated with lipid synthesis, transport, and storage: fatty acid synthase (FAS), acyl-CoA synthetase-1 (ACS1, fatty binding protein-4 (FABP4, fatty acid transport protein-1 (FATP1), and perilipin [126]. The mushroom supplements also induced AMP-activated protein kinase (AMPK) and increased glucose uptake by the adipocytes. It seems that the *Ganoderma lucidum* formulation has multiple beneficial effects on transcription factors and genes that induce adipocyte differentiation, synthesis, transport, and storage of lipids associated with obesity and diabetes.

Other observations of the beneficial properties of *Ganoderma lucidum* suggest that the properties might be largely associated with polysaccharide components. For example, Chang, et al. [98] showed that a water extract of the Chinese medicinal mushroom *Ganoderma lucidum* mycelia reduced body weight, inflammation, and insulin resistance in mice fed a high-fat diet. The mechanism of the beneficial effects seems to involve reversal of HFD-induced gut dysbiosis as indicated by the *Firmicutes*/*Bacteroides* ratio and endotoxin-containing *Proteobacteria* levels in the gut, maintenance of intestinal barrier integrity, reduced endotoxemia, reduced expression of genes involved in fatty acid synthesis, and insulin resistance and signalling pathways affecting resistance. Similar antiobesity and microbiota-modulating effects were observed with isolated high-molecular weight (>300 kDa; 47.5% mannose, 26.3% glucose, and 16.9% galactose) polysaccharides, suggesting that the antiobesity effect of the extract might be largely associated with the polysaccharide. This comprehensive study also implies that the extract and isolated polysaccharide might serve as prebiotics to reduce obesity, inflammation, and insulin resistance in humans.

A purified polysaccharide isolated from an aqueous extract of *Ganoderma lucidum* mushroom (also known as Lingzhi or Reishi medicinal mushrooms) fruit bodies was injected into mice to assess its safety [127]. The results showed that the injection mixture improved antifatigue symptoms of the mice and was not allergic in a guinea pig test, suggesting a novel approach to studies of health-promoting properties of this and possibly other polysaccharides.

#### 5.1.4. Pleurotus Tuber-Regium

Because obesity and hyperglycemia are closely linked syndromes associated with increased serum cholesterol and triglyceride levels, it was of interest to determine if mushroom polysaccharides can ameliorate both syndromes. This seems to be the case for *Pleurotus tuber-regium* because oral administration (20 mg/kg body weight/8 weeks) of extracted exo-polysaccharides preparations with different molecular weights from submerged culture media of the edible mushroom to obese-diabetic rats ameliorated both obesity and hyperglycemia [122,123]. The beneficial effects were accompanied by the following changes in associated biomarkers: (a) attenuation of fatty acid component *n*-6/*n*-3 ratio in liver and plasma; (b) controlled serum total cholesterol, triglycerides, and low-density lipoprotein (LDL) and decreased high-density lipoprotein (HDL) levels; and (c) restored decreased fasting insulin, monounsaturated to polyunsaturated (MUFA/PUFA) and monounsaturated to saturated (MUFA/SFA) fatty acid ratios, and adiponectin levels. The mechanism of the anti-lipidemic effect seems to be associated with the up-regulation of lipid metabolism by the liver peroxisome proliferator-activated receptor-alpha (PPAR-α) and m-RNA expression. These polysaccharides have the potential to be used as a dietary supplement to treat HFD-induced metabolic syndrome. Surprisingly, the cited studies show that only a few mushroom polysaccharides have been evaluated for their potential to ameliorate obesity.

The following additional selected studies provide a starting point for further reading on the antiobesity properties of mushroom polysaccharides and mushrooms: Mizutani, et al. [128]; Handayani, et al. [129]; Inoue, et al. [130]; Kanagasabapathy, et al. [131]; Vincent, et al. [132]; Handayani, et al. [133]; Park, et al. [134]; Zheng, et al. [135]; Iuchi, et al. [136]; Mori, et al. [137]; and Reza, et al. [138].

In relation to this, there is extensive literature on the antiobesity properties of cereal β-glucans (for example, from barley, oats, rice, wheat) in rodents and humans that may be concurrently consumed with corresponding mushroom β-glucans: for example, Aoe, et al. [139]; Arena, et al. [140]; Chang, et al. [141]; El Khoury, et al. [142]; and Tabesh, et al. [143].

### 5.2. In Humans

So far, there are relatively few studies that report on the antiobesity properties of mushrooms in humans that may be associated with their content of polysaccharides.

**Figure 7:**
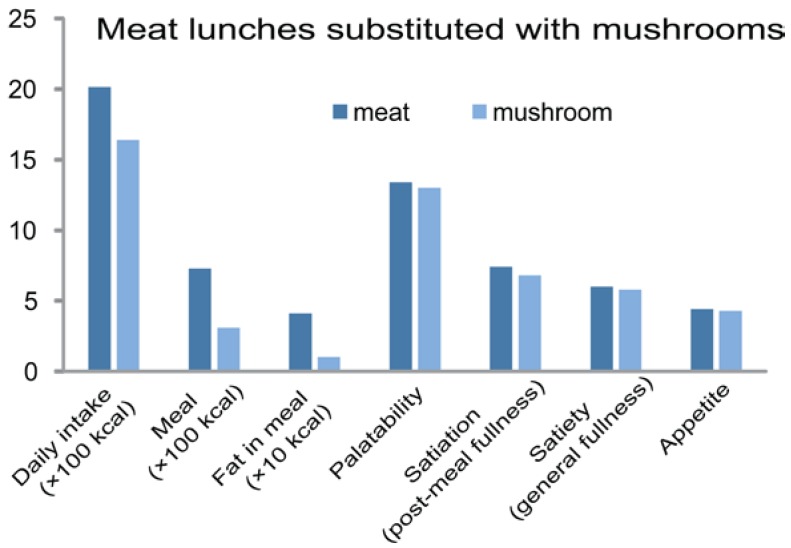
A mushroom-containing reduced calorie meal served over 4 days did not negatively affect satiety. Adapted from Cheskin, et al. [144].

A short-term 4-day clinical human trial with men and women with obesity or type-2 diabetes was designed to determine the effect of replacing 20% of high-energy lean ground beef in the diet with 20% of low-energy ground white button mushrooms (*Agaricus bisporus*) [11,144]. Figure 7 shows that substituting meat with lower energy mushroom-containing lunches with energy values of 783 and 339 kcal, respectively, reduced daily energy and fat intake and did not negatively affect palatability, appetite, and satiety.

**Figure 8:**
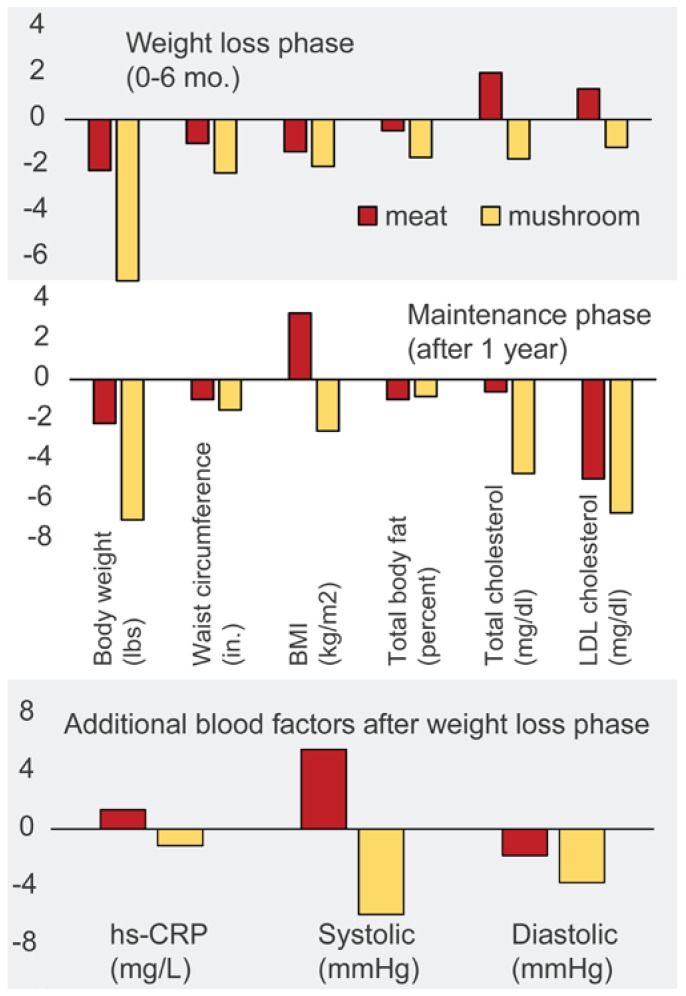
Weight loss diet substituting white button mushrooms for beef with no change in caloric content. Based on data from Poddar, et al. [11].

A more extensive long-term 1-year clinical human trial with overweight men and women showed that individuals consuming an isocaloric mushroom diet, relative to a meat diet, lost weight, had lower blood pressure and plasma lipid (total and LDL cholesterol, triglycerides), fasting glucose levels, and reduced levels of the inflammation biomarker hs-CRP associated with obesity (Figure 8) [11].

In addition, participants consuming the mushroom diet had lower BMI (basal metabolic index), lower calorie (energy) and fat intakes, reduced waist circumferences, high satiety without affecting palatability than those on the meat diet. The authors suggest that simply substituting one food for another may be more appealing to dieters than more restrictive changes in dietary behavior. These studies merit extension to other mushroom varieties, especially to the highly bioactive and health-promoting *Hericium erinaceus* (Lion’s mane) and *Lentinus edodes* (shiitake) varieties [22,31]. Will mushroom polysaccharides induce similar beneficial effects in humans?

A related study found that adding crimini or white mushrooms (*Agaricus bisporus*) into a beef taco blend enhanced the flavor of the reduced salt version of the tacos, suggesting that the flavor-enhancing umami principles of mushrooms can be used as a health substitute for meat that also mitigates sodium reduction without loss of overall flavor [145]. It is also noteworthy that the addition of 4% β-glucan-rich fractions from *Pleurotus eryngii* mushrooms to wheat flour improved the quality of wheat pasta and resulted in hardness similar to those of semolina pasta [146], suggesting that consumers might favor such pastas for both quality and health benefits. Moreover, an assessment of the relationships of dietary patterns to the risk of obesity shows that the so-called ‘Healthy’ pattern is characterized by a high intake of mushrooms, potatoes, seaweeds, fish, shellfish, and soy products [147].

## 6. Antidiabetic Properties

### 6.1. Historical Perspective on the Cause and Treatment of Diabetes

Roberts [148], offers a fascinating historical account of the evolution of our understanding of the cause and treatment of diabetes that includes the following aspects: (a) At around 1550 years Before the Christian Era (BCE), the first recorded unsuccessful treatment for ‘excessive urination’ now associated with diabetes appeared in the Egyptian Ebers Papyrus, a medical papyrus; (b) About 1500 years later, the Greek physician Aretaeus coined the term diabetes (siphon) in the first century CE to describe excess siphoning of water into the urine; (c) In 1776, the English physician Thomas Willis rediscovered the connection between diabetes and sweetness of the urine, now known to be due to the presence of glucose, and renamed the disease ‘diabetes mellitus’ (like honey); (d) In 1889, the physiologist Oskar Minkowski at the University of Strasbourg was the first to associate the pancreas with diabetes, following his discovery that a dog developed diabetes after he removed the pancreas; and (e) In 1921, the Canadian physician Frederick Banting and his assistant Charles Best isolated the protein insulin from the pancreas of a dog and successfully used it to treat the 14-old diabetes patient Leonard Thompson, thus continuing the still elusive search for a complete cure.

### 6.2. Antidiabetic Activities of Mushroom Polysaccharides

**Figure 9:**
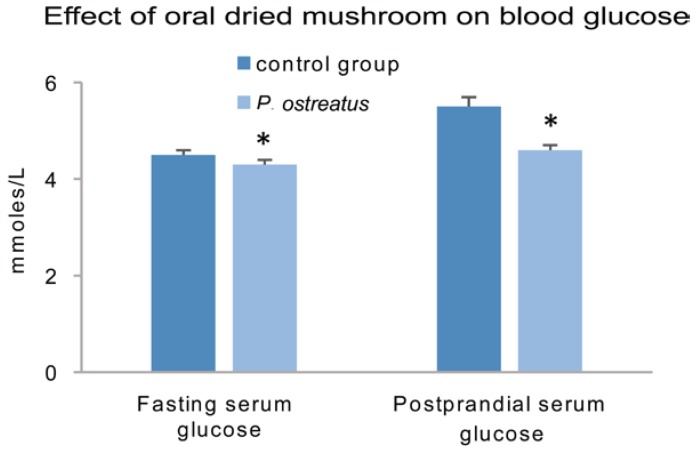
Orally administered dried and powdered Oyster mushrooms (50 mg/kg BW/day) for 2 weeks improved glucose control in type-2 diabetic humans. Adapted from Jayasuriya, et al. [167].

**Figure 10:**
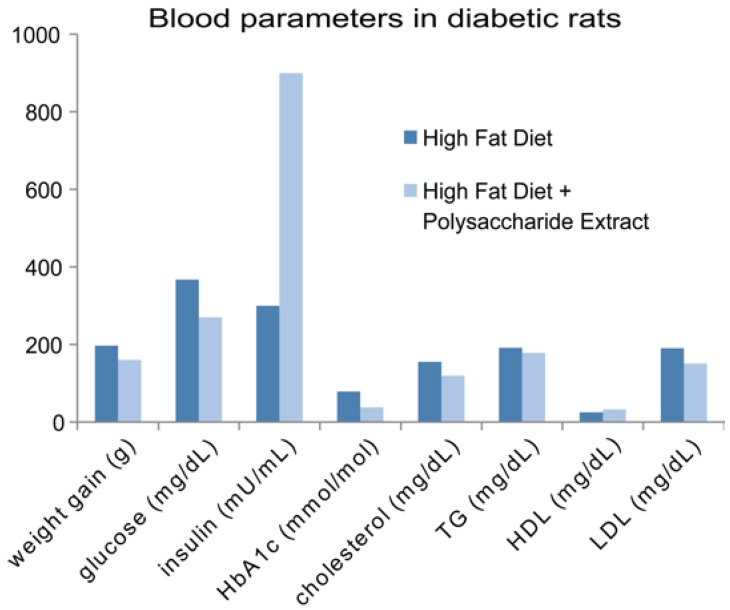
Addition of *Pleurotus tuber-regium* polysaccharide extract to a high fat diet in streptozotocin-induced diabetic rats improved blood parameters and was accompanied by reduced hepatic lipid peroxidation and increased hepatic superoxide dismutase and glutathione peroxidase activity and peroxisome proliferator activated receptor (PPAR)-α expression. Adapted from Huang, et al. [122].

High-fat diets (HFD) increase plasma glucose levels and decrease insulin secretion in healthy individuals (Figure 9) [149,150]. A major feature of insulin resistance that accompanies diabetes includes dyslipidemia, characterized by high triglyceride, low high-density lipoprotein (HDL), and high low-density lipoprotein (LDL) serum levels [151,152]. Biomarkers (Figure 10) in the serum liver, gut and pancreas that may be associated with the mechanisms of antidiabetic agents include: control of serum glucose and lipoproteins; beneficial effects on immunity; increase in antioxidative enzyme activities in serum; increase in serum insulin; influence on gut microbiota; changes in liver PPAR-α expression and protein levels; and structural changes in pancreatic β-cells. Mushroom polysaccharides are reported to lessen the symptoms of medical conditions associated with metabolic syndrome, including diabetes [153]. Polysaccharides that have been reported to have antidiabetic properties in diabetic and/or obese rodents include those from the following mushroom varieties: *Agaricus brasiliensis* [154]; *Agrocybe chaxingu* [155]; *Catathelasma ventricosum* [18]; *Grifola frondosa* [156]; *Phellinus linteus;* [56]; *Pleurotus abalonus* [157]; *Pleurotus eryngii* [158]; *Pleurotus florida* [159]; *Pleurotus sajor-caju* [160]; *Tremella fuciformis* [151]; *Ganoderma lucidum* [161]; *Lentinus strigosus* [162]; *Pleurotus tuber-regium* [123]; *Sparassis crispa* [163]; and *Tremella aurantialba* [164,165,166]. Several of these studies will be elaborated on further below.

### 6.3. Mechanisms of Bioactivities of Polysaccharides against Diabetes

Macrophages in the mucus lining of the intestine bind β-glucans through β-glucan receptors [20,168]. These cells are then activated and move back to the lymph nodes (Payer’s Patch) that then release cytokines and induce immune activation. β-Glucans also prevent absorption of cholesterol from food in the digestive tract, thus lowering serum cholesterol. It seems that a β-glucan-containing fraction from *Agaricus blazei* was more effective in reducing plasma sugar levels in rats than pure β-glucan from barley. Lei, et al. [169] discovered that an α-glucan from *Grifola frondosa* protected murine pancreatic cells against oxidative damage.

A polysaccharide from oyster mushrooms at a concentration of 0.4 mg/mL inhibited the enzyme α-glucosidase with an IC50 value of 0.424 mg/mL [38]. Because α-glycosidase enzymes are known to induce postprandial hyperglycemia and other maladies, this result suggests that glycosidase-inhibiting mushroom polysaccharides might be of value in therapy for diabetes.

The oral administration of extracellular polysaccharides (20 mg/kg body weight/8 weeks) from three strains of *Pleurotus tuber-regium* mushrooms to obese diabetic rats attenuated both obesity, by maintaining a stable fatty acid composition, and hyperglycemia. The hypolipidemic properties were accompanied with up-regulated liver PPAR-α mRNA expression and protein levels [123]. The polysaccharides seem to act as PPAR-α agonists that result in decreased serum triglyceride and increased HDL levels by increasing lipid uptake, activation, and catabolism via transcriptional changes of genes that control these events. Duobin, et al. [170] observed similar hypolipidemic properties of exopolysaccharides produced by *Pleurotus geesteranus* fermented mushrooms in diabetic mice.

Detailed studies by Kanagasabapathy et al. [132,160,171] showed that polysaccharide-rich β-glucans (240 mg/kg body weight) from *Pleurotus sajor-caju* mushrooms prevented the occurrence of hyperglycemia and hyperinsulinemia/insulin resistance in mice fed a high fat diet by upregulating the expression of insulin-response glucose-transporter (GLUT-4) and the antidiabetic, antiatherogenic hormone adiponectin genes and down-regulating the expression of nuclear factor κB (NF-κB) that controls the regulation of genes that encode proteins involved in immune and inflammatory responses, including interleukin-6 (IL-6). The amelioration of insulin resistance was similar to that observed with the antidiabetic drug metformin.

These authors also discuss the factors that might govern the mechanisms of causes and prevention of diabetes at the molecular–genetic levels, including the following events. Diabetes causes a reduction in the production of anti-inflammatory adiponectin and an increase in the production of pro-inflammatory cytokines. The inflamed state leads to the release of cytokines in adipose tissues that are not usually secreted by adipose cells. Thus, the expression of inflammation tumor necrosis factor-α (TNF-α) and IL-6 amplifies insulin resistance in the adipose tissues of HFD animals. This suggestion is supported by the results, where the HFD groups had higher expression of the pro-inflammatory biomarkers compared to the polysaccharide-treated groups. By contrast, the expression of NF-κB in the HFD groups treated with polysaccharide and metformin was significantly downregulated; the levels were lower than those of the untreated HFD group [171].

Studies of mechanistic interest showed that the same exopolysaccharide induces lipogenesis in differentiating 3T3-L1 cells and in lipolysis in 3T3L-1 adipocytes [160]. This mechanism seems to depend in part on the ability of the exopolysaccharide to upregulate the gene expression of adiponectin and leptin hormones produced by adipose tissues and of GLUT-4, and downregulate the inflammatory biomarkers through activation of the AMPK-signalling pathway. These events, associated with a novel AMPK activator that acts as a regulator of glucose and lipid homeostasis in adipocytes, might help formulate functional food for the prevention and treatment of diabetes.

Treatment of diabetic mice with α-glucan (150 or 450 mg/kg) from *Grifola frondosa* mushrooms decreased body weight, plasma glucose, glycosylated serum protein, serum insulin, triglycerides, cholesterol, free fatty acids, and malondialdehyde levels in livers [172]. The treatment also increased the content of hepatic glycogen, reduced glutathione, and the activities superoxide dismutase, and glutathione peroxidase enzymes, suggesting that the multiple beneficial biochemical changes might be due to the effect of the α-glucan on insulin receptors that results in increased insulin sensitivity and ameliorated insulin resistance of peripheral target tissues.

A related study [156], reported that the antidiabetic effect of an α-glucan (100 or 300 mg/kg body weight) from the fruit body of *Grifola frondosa* mushrooms on mice on a high-fat diet was associated with improved (adjusted) immune function and its effect of decreasing the levels of factors that destroy β-pancreatic cells. Indeed, the treatment decreased the levels of fasting plasma glucose, triglycerides, cholesterol, free fatty acids, and NO production by the macrophages, effectors that cause immune disruption. It also increased serum insulin level and beneficially affected production of immunocytokines by splenocytes that play a key role in immune responses, and ameliorated ultrastructural changes in pancreatic β-cells, suggesting that the observed hypoglycemic and hypolipidemic effects on the diabetic mice might be related to parallel beneficial effects on immune reactions involved in pathogenesis of diabetes mellitus.

Two protein-bound exopolysaccharides isolated from mycelial cultures of *Phellinus baumii* (consisting of mannose, 56.1%; xylose, 21.7%; and fucose, 19.6%; and carbohydrate and protein proportions of 71.0% and 29.0%, respectively) and *Tremella fuciformis* mushrooms (consisting of 87.5% mannose, and 7.0% galactose in the carbohydrate chain), widely used as folk medicines, significantly reduced fasting blood glucose levels and improved glucose tolerance without affecting body weight gain of ob/ob (obese) mice [151]. The observed activation (increase) of PPAR-γ transcription messenger expression in response to the treatment seems to be related to the regulation of hyperglycemia and dyslipidemia, possibly via the regulation of lipid metabolism.

Treatment of diabetic rats for 7 days with a crude exopolysaccharide (50–150 mg/kg body weight) from a submerged mycelial culture of the edible *Lentinus strigosus* mushroom induced a dose-dependent, up to 21.1% reduction in the serum glucose level, hypoglycemia, and regeneration in the pancreatic islets of Langerhans and amelioration of the destruction of microvasculature of the islets [162].

Because abnormal lipid metabolism and increased oxidative stress are major complications of diabetes, Zhang, et al. [164] evaluated the potential of a saponin-containing broth extract and polysaccharides from *Tremella aurantialba* mycelia to alleviate these conditions in diabetic rats. The extract was more effective than the polysaccharides in decreasing blood glucose, cholesterol, phospholipids, and triglyceride serum levels. The extract was also more effective in inducing increase in superoxide dismutase, catalase, glutathione peroxidase and reductase levels that are associated with oxidative stress, suggesting that the metabolic profile (mechanism) that regulates the action of mushroom saponins seems to differ from that of the polysaccharides. It would be interesting to determine if the combination of the two bioactive mushroom formulations (saponins and polysaccharides), as well as terpenoid- and sterol-rich constituents of an extract of *Inonotus obliquus* medicinal mushrooms with hypoglycemic activity [173], act additively or synergistically against diabetes.

An investigation by Lee, et al. [155] showed that a polysaccharide from *Agrocybe chaxingu* mushrooms prevented the destruction of pancreatic β-cells in mice. The beneficial effect was accompanied by reduced plasma glucose and nitric oxide (NO) production and inducible nitric oxide synthase (iNOS) expression in the cells in a dose-dependent manner, suggesting the value of the polysaccharide to treat diabetes mellitus.

A 120-kDA polysaccharide isolated from the fruiting bodies of *Pleurotus abalonus* (composed of glucose, rhamnose, glucuronic acid, xylose, galactose, and arabinose in the molar ratio of 26.3:2.7:1.1:1.4:1.8:1.2) exhibited hypoglycemic activity in diabetic mice and inhibited MCF7 breast cancer cells with an IC50 of 3.76 μM and HIV-1 reverse transcriptase with an IC50 of 8.7 × 10^−2^ μM [157]. These observations suggest that this polysaccharide might alleviate diabetes, cancer, and HIV infections in humans.

Huang et al. [122,123] found that the administration of *Pleurotus tuber-regium* mushroom polysaccharides to diabetic rats on a high-fat diet resulted in reduced fasting blood glucose and glycosylated hemoglobin (HbA1c) levels and restored serum insulin levels, and restoration of total cholesterol, triglycerides, LDL and HDL levels. The polysaccharides inhibited the lipid peroxidation index (malondialdehyde) and restored oxidative enzymes (superoxide dismutase and glutathione peroxidase) activities in the liver. The authors suggest that the antioxidative property of the polysaccharides might enhance the antioxidant system that attenuates oxidative stress, indicating that the polysaccharides might be an alternative medicine to treat hyperglycemia and oxidative stress associated with diabetes.

Homogeneous water- and alkali-extracted polysaccharides from *Pleurotus tuber-regium* mushrooms exhibited strong antioxidative properties in multiple antioxidative chemical and cell assays (oxygen and hydroxyl radical and DPPH scavenging activities; inhibition on liver peroxidation, liver mitochondria swelling, and red blood cell hemolysis) [174]. The water-extracted polysaccharide was more effective than the alkali-extracted one. As mentioned earlier, another study found that oral administration of extracellular polysaccharides (20 mg/kg body weights/8 weeks) from three different strains of *Pleurotus tuber-regium* mushrooms to obese-diabetic rats alleviated molecular and cellular biomarkers associated with both obesity and diabetes [123]. The cited studies imply that the polysaccharides are potent natural antioxidant that might alleviate oxidative damage in food and oxidative stress in vivo associated with diabetes, obesity, and other diseases.

The administration of a mixture of polysaccharides from *Catathelasma ventricosum* mushrooms, a traditional Chinese medicine, to diabetic mice for 30 days results in reduced blood glucose, total cholesterol, triglycerides, and LDL-cholesterol, and an increase in HDL cholesterol and antioxidant enzymes [18]. The beneficial properties also seem to be related to potent antioxidative activities of the polysaccharides. Because pathologic morphologies of the liver, kidney, and pancreas of treated diabetic mice were similar to those of normal mice, it also seems that the polysaccharides are safe compounds. It is relevant to note that selenium-enriched mycelia of this mushroom exhibited antihyperglycemic and antioxidant properties that seem to be caused by a selenium-containing protein with the amino acids selenocysteine and selenomethionine [33].

A polysaccharide isolated from the medicinal mushroom *Phellinus linteus* mycelia grown in submerged culture was mainly a branched-type glycan with both β- and α-linkages and a pyranoid sugar conformation, as determined by FT-IR and NMR spectroscopies [56]. The polysaccharide was isolated under the following optimum conditions: 90 °C with a 1:50 ratio of mycelia to hot water and a precipitation time of 2 h. Oral administration of the polysaccharide (100 mg/kg body weight/day) to diabetic mice significantly reduced the blood glucose level by 35.6%, suggesting the value of this mushroom compound as a functional food additive and a hypoglycemic agent.

A heteropolysaccharide isolated from *Pleurotus eryngii* mushrooms consisted of the following monosaccharides (in mole %): d-glucose (62.8), d-galactose (24.4), and d-mannose (9.8). Administration of the polysaccharide to mice at 400 and 800 mg/kg body weight significantly reduced the serum fasting glucose and insulin levels and lipid deposition in high-fat mice [175]. It also induced reduction of liver lipid peroxidation and the elevation of the hepatic antioxidant system. The histopathological examination of the liver further confirmed the hepatoprotective effect. The authors suggest that the polysaccharide has the potential to mitigate insulin resistance, oxidative stress, and liver dysfunction. The results of a related study on the effect of an extract of the same mushroom injected into diabetic mice suggested the action of the extract on glycemic metabolism occurs via increasing glycogen and insulin levels, reducing free radical damage, and recovering injured pancreatic β-cells [158].

A polysaccharide isolated from the culinary–medicinal *Pleurotus florida* mushrooms was found to be nontoxic to mice up to 4000 mg/kg body weight [159]. The treatment of diabetic mice with the polysaccharide (200 and 400 mg/kg) resulted in significant reduction of the plasma glucose, serum cholesterol, triglycerides, malondialdehyde, nitric oxide, and urine glucose and ketones concentrations. By contrast, the concentrations in the liver of superoxide dismutase, catalase, and reduced glutathione were restored to normal levels, suggesting that the polysaccharide might ameliorate hyperglycemia and hypocholesteremia associated with diabetes.

In a further analysis of the mechanism of action of polysaccharides in diabetes, Karumuthil-Melethil, et al. [176] reported that immune cells recognize β-glucans through a cell surface pathogen recognition receptor called dectin-1, suggesting that the observed innate immune response induced by low-dose β-glucan is regulatory in nature that can be used to modulate T cell response to pancreatic β-cells for inducing protection from type 1 diabetes (T1D), where the pancreas does not produce any insulin. For additional details on the relationship of β-glucans to the dectin-1 signalling pathway, see the review by Lee and Kim [177].

The following observations do not directly involve the use of polysaccharides, but are relevant to the theme of the review. A submerged culture of mycelia and broth of the medicinal mushroom *Grifola frondosa* alleviated type 2 diabetes by decreasing cell-mediated immunity in normal rats and improved hyperglycemia in diabetes-induced innate immunities [178]. Administration of a 70% ethanol extract of the medicinal mushroom *Auricularia auricula-judae* to obese mice reduced the risk of hepatic steatosis by modulating plasma lipids via the regulation of adipogenic and lipogenic transcriptional factors, suggesting the value of the extract to improve plasma lipids and liver enzymes [138].

A retrospective study of 37 human subjects indicates that dietary consumption of *Agaricus bisporus* mushrooms reduces diabetes risk factors, suggesting that the mushrooms contain compounds with potential anti-inflammatory and antioxidant health benefits that can occur over time in adults predisposed to type-2 diabetes [179]. Related observation show that the *Agaricus bisporus* mushroom had both hypoglycemic and hypolipidemic activity in rats [180], and that *Agaricus bisporus* lectins (glycoproteins) regenerated pancreatic β-cells in mice following 70% partial pancreatectomy, suggesting that induction of islet β-cell proliferation has therapeutic potential for diabetes [181].

### 6.4. Polysaccharides Accelerate Wound Healing in Diabetic Rats

Wound healing is delayed and impaired in diabetic individuals. To help overcome this problem, two studies have described the benefits of polysaccharide-containing mushrooms. Cheng, et al. [161] found that topical application of an aqueous extract of *Ganoderma lucidum* mushrooms containing 25.1% polysaccharides incorporated into a cream was effective in healing diabetic rats wounded in the posterior neck region. Additional observations showed that the antioxidant activity in the serum of treated rats was higher, the oxidative damaged lipid and protein content of the serum was significantly lower, and the collagen matrix with newly formed capillaries of the wounds was higher than those of the controls, suggesting the potential value of the mushroom formulation in wound management that could avoid the need to amputate affected organs of diabetic individuals. A related study reported that oral administration of the medicinal mushroom *Sparassis crispa*, with a β-glucan content of 40.5%, accelerated skin wound closure of diabetic rats [163]. The improvement in healing seems to involve migration of macrophages, fibroblasts, and β-glucan that leads to increases in the synthesis of type I collagen. The cited observations merit confirmation with wounds of diabetic humans.

### 6.5. Human Clinical Studies on the Efficacy of Mushrooms against Diabetes

Only a limited number of studies have been conducted in humans on the beneficial properties of mushroom polysaccharides.

An evaluation of the effect of the culinary *Pleurotus ostreatus* and *P. cystidiosus* mushrooms (consumed as freeze-dried powders at a dose of 50 mg/kg/body weight) in healthy human volunteers and type 2 diabetic patients showed: (a) a reduction in fasting and postprandial serum glucose levels of healthy volunteers; and (b) a reduction in postprandial serum glucose levels and increased serum insulin levels of type 2 diabetic patients [167]. The mechanism of the hypoglycemic activity seems to be associated with increased glucokinase activity and promotion of insulin secretion by the pancreas, thus increasing the utilization of glucose by peripheral tissues, inhibiting glycogen synthase kinase activity, and promoting glycogen synthesis. It is also worth noting that evidence from a limited number of trials does not support the use of *Ganoderma lucidum* mushrooms for the treatment of risk factors of cardiovascular disease (blood glucose level, blood lipid profile, blood pressure) in individuals with type 2 diabetes [182]. A journal reviewer suggested that if experiments were conducted with impure polysaccharides or mushroom extracts, it is likely that other bioactive compounds could have contributed to the antidiabetic activities.

Because diabetes is often accompanied by symptoms of depression [183], Nanri [184], carried out an epidemiological study over a 5-year period with 55,000 Japanese men and women aged 45 to 75 years designed to help define the role of the diet in influencing the incidence of both diabetes and depression symptoms. The results suggest that type-2 diabetes increased with rice intake among women and physically inactive men and decreased with consumption of seafood among men and with soy products among overweight and postmenopausal women. With respect to accompanying depression, the results revealed an inverse association with serum folate and vitamin D levels and intake of fruits, vegetables, isoflavone-containing soy products, and mushrooms, suggesting that diet affects the development of both diabetes and depression and that consumption of commercially available high-vitamin D mushrooms might ameliorate the incidence of depression. Although this is a more general study on diet, diabetes, and depression that does not directly involve polysaccharides, it gives an indication of how dietary supplementation can alleviate such associated conditions.

## 7. Anticancer Effects

Biochemical and cellular biomarkers associated with diabetes and obesity are also operative against different types of cancers and infections, suggesting the possibility that mushrooms and their polysaccharides might concurrently protect against multiple human diseases. Such biomarkers include antiradical, antioxidant, anti-glycation [185], anti-inflammatory [168], and anti-α-glucosidase [38,186], activities and enhancement of the immune system [47,177]. To stimulate interest in this possibility, here we will briefly mention selected studies on the anticarcinogenic and antibiotic properties of mushrooms.

A seminal study describes the development in Japan of three different polysaccharide agents [187]. This paper includes illustrations of a three-dimensional molecular model of the right-handed triple spiral helix of an active β-d-glucan. A meta-analysis of 8009 patients from randomized controlled trials revealed that addition of the immunopotentiator polysaccharide K (PSK) from *Coriolus versicolor* mushrooms to standard chemotherapy increased the survival of patients after curative gastric cancer resection over chemotherapy alone [188,189]. This study confirms the ability of polysaccharides to induce apoptosis and other forms of cancer cell death through immunological mechanisms [190]. It is also noteworthy that daily consumption of *Lentinula edodes* improved immunity in healthy young adults and that oral consumption of soluble β-glucans was safe in elderly healthy adults and seemed to induce an increase in the number of circulating β-cells [191].

Cell, animal, and human studies indicate that blended (combination of extracts from three different mushrooms) extracts exhibit significantly stronger cytotoxic effects on human tumor cell lines than single extracts [192,193].

A journal reviewer noted that maybe the best solution in practical use would be blended mushroom liquid extracts that contain active antitumor polysaccharides and a large number of other bioactive compounds such as flavonoids and triterpenes. For example, *Hericium erinaceus* mushrooms contain about 50 characterized secondary metabolites [22]. As noted above, it might be possible to increase the biosynthesis of polysaccharides by genetic engineering methods. It might also be worthwhile to determine the bioactivities of three different polysaccharides present in a hybrid mushroom obtained through backcross mating of a somatic hybrid mushroom through protoplast fusion [194], as well as the contribution to bioactivity of the extracted polysaccharides and the non-extracted part that contains bioactive compounds.

The efficacy of mushrooms and their bioactive components against cancer are reviewed in Ren, et al. [51] and briefly outlined here:

1. Dietary supplementation of *Agaricus silvaticus* mushrooms reduced glycaemia levels of post-surgery cancer patients [195];
2. Oral administration of a polysaccharide from *Grifola frondosa* stimulated the immune system of breast cancer patients [196];
3. Dietary supplementation with *Agaricus silvaticus* provided metabolic and blood pressure benefits to postsurgical colorectal cancer patients [197];
4. Several Japanese studies reported that orally administered *Lentinula edodes* mycelia extracts improved the course of cancer in Japanese patients undergoing chemotherapy [198,199,200,201,202];
5. The mushroom β-glucan lentinan prolonged the survival of patients with advanced gastric cancer [203];
6. Dietary administration of *Agaricus silvaticus* mushrooms improved nutritional status and reduced abnormal bowel functions and nausea in patients undergoing chemotherapy for breast cancer [204];
7. A meta-analysis suggests that dietary mushroom intake seemed to be inversely associated with risk of breast cancer [205];
8. Oral consumption of mushrooms glucans seems to be an efficient treatment to prevent colitis-associated cancer through modification of mucosal inflammation and cell proliferation [206];
9. Consumption of an *Agaricus bisporus* mushroom powder by prostate cancer patients beneficially impacted prostate-specific antigen (PSA) levels and modulated the biology of recurrent prostate cancer by decreasing immunosuppressive factors [207].

The following additional observations further highlight the efficacy of polysaccharides to contribute to the prevention and therapy of cancer. Combination therapy using a β-glucan fraction from *Grifola frondosa* and cytosine-phosphate guanine oligodeoxynucleotide was highly effective, both as an adjuvant for dendrite cell vaccination and by direct administration, against a murine tumor, suggesting that the combination has potential as an immunotherapeutic approach against cancer [208]. A related study by Pan, et al. [209] showed that a polysaccharide from the medicinal mushroom *Amauroderma rude* inhibited tumor growth in mice via regulation of the immune system at the molecular and cellular levels. Encapsulation of polysaccharides from the medicinal mushroom *Antrodia camphorata* in chitosan-silica or silica nanoparticles enhanced anti-tumor efficacy of HepG2 liver cancer cells [210]. Polysaccharide-containing *Hericium erinaceus* mushroom extracts prevented migration of cancer cells of implanted colon tumors in mice to the lung via targeting their upstream signalling molecules for mediating the expression of the extracellular matrix-degrading proteinases [211].

It is also noteworthy that daily consumption of *Lentinula edodes* improved immunity in healthy young adults and that oral consumption of soluble β-glucans was safe in elderly healthy adults and seemed to induce an increase in the number of circulating β-cells [191]. Relevant studies by Meng, et al. [69] and Yan, et al. [70] discuss in detail the relationship between structural features of mushroom polysaccharides and antitumor properties Schwartz and Hadar [206] discuss possible mechanisms of action of orally administered (but poorly bioavailable) mushroom β-glucans against human cancer associated with inflammatory bowel disease, suggesting the need for clinical evidence on the beneficial anticarcinogenic activities of mushrooms and their polysaccharides in humans [212].

### 7.1. Anti-Aromatase Activity against Breast Cancer

Santen, et al. [213] hypothesized that by catalyzing the conversion of androgens to cancer-causing estrogens, the enzyme aromatase seems to contribute to the cause of breast cancer and that aromatase inhibitors would inhibit the process of tumor promotion by lowering tissue levels of estradiol and thus blocking cell proliferation. Studies with mushrooms support this possibility. Thus, Grube, et al. [214] found that *Agaricus bisporus* phytochemicals modulate aromatase activity and have the potential to chemoprevent breast cancer in postmenopausal women by reducing the in-situ production of estrogen. Phytochemicals in white button mushrooms suppressed aromatase activity in the MCF-7 breast cancer cell line in vitro and in hamsters resulting in both decreased tumor cell proliferation and tumor weight [215]. It is not known if polysaccharides contribute to these beneficial effects.

### 7.2. Crohn’s Disease Therapy

Crohn’s disease is an incurable chronic inflammatory human disease that affects the distal ileum and colon and other parts of the GI tract causing chronic diarrhea and abdominal pain [14]. Male patients show an increased risk of development of colo-rectal cancer and all cancers compared with controls [216]. Treatments include the use of immunomodulators. Norwegian scientists reported that ingestion of an *Agaricus blazei* mushroom extract AndoSan™ improved symptoms in patients suffering from Crohn’s disease and the related malady ulcerative colitis [217,218]. The authors suggest that because the treatment did not seem to cause any adverse effects, the mushroom preparation has the potential to be used as a safe supplement to conventional medication. Additional studies revealed that the effect of the treatment on reduction in systemic cytokine levels was limited, suggesting that the mechanisms that might govern the clinical anti-inflammatory effect as well as the possible role of polysaccharides [219,220] merit further study.

## 8. Antimicrobial Activities

The following selected observations indicate that mushroom polysaccharides and derivatives exhibited strong antibiotic properties against pathogenic bacteria and viruses.

### 8.1. Antibiotic Effects

Supplementation with the β-glucan from *Pleurotus ostreatus* might protect athletes against infections of the respiratory tract [221]. Silver nanoparticles prepared using glucan isolated from *Pleurotus florida* blue variant mushrooms inhibited the multiple antibiotic-resistant (MAR) bacterium *Klebsiella pneumoniae* in a dose-dependent manner and acted synergistically with four medicinal antibiotics to inhibit nearly all bacterial growth, suggesting that the combinations might control the MAR bacteria that cause pneumonia [222]. A related study by Manna, et al. [62] reported that nanoparticles synthesized using a hetero polysaccharide isolated from *Lentinus squarrosulus* mushrooms exhibited antibacterial activity against MAR *E. coli* bacteria and exhibited synergistic effects with four antibiotics to inhibit all bacterial growth. It seems that the nanoparticles are more effective than normal-sized particles in penetrating and destroying bacteria and viruses.

The *Lentinus edodes* polysaccharide protected mice against a *Salmonella* lipopolysaccharide-induced endotoxemia (septic shock), an often-fatal human disease [31]. The same polysaccharide and a *Hericium erinaceus* mushroom extract protected mice against infections by the lethal foodborne pathogen *Salmonella* Typhimurium via stimulation of the immune system [92,93]. A purified *Lentinus edodes* extract exhibited antimicrobial properties against oral bacterial pathogens, suggesting their value in the improvement of oral hygiene [223]. A crude polysaccharide from *Auricularia auricula-judae* showed in vitro activities against the foodborne pathogens *Escherichia coli* and *Staphylococcus aureus* [224]. A sulphated polysaccharide from *Pleurotus eryngii* (oyster) mushrooms inhibited the foodborne *E. coli* and *Staphylococcus aureus* pathogenic bacteria [225].

### 8.2. Anti-Quorum Sensing Mushroom Compounds

Biofilm is a slimy layer that can surround (occlude) bacteria and confer resistance to phagocytosis and antibiotics [14]. De Carvalho, et al. [226] isolated the compound coprinuslactone [(3R,4S)-2-methylene-3,4-dihydroxypentanoic acid 1,4-lactone] from the edible mushroom *Coprinus comatus* that interfered with quorum sensing and dispersed biofilms of *Pseudomonas aeruginosa*, where it also reduced the formation of pathogenicity factors pyocyanin and rhamnolipid B. In addition, coprinuslactone also damaged *Staphylococcus aureus* cells in biofilms at subtoxic levels and inhibited UDP-acetyl glucosamine enolpyruvyl transferase that is essential for cell wall synthesis. Related studies showed that extracts of *Agaricus* mushroom species inhibited the biofilm forming ability of *P. aeruginosa* [227], and that extracts of *Ganoderma lucidum* and *Phellinus ignarius* inhibited quorum sensing in *Chromobacterium violaceum* [228]. These observations suggest that mushroom produce compounds that can serve as a source of antimicrobial and anti-quorum sensing agents. Will mushroom anti-quorum sensing compounds inactivate pathogens in vivo via stimulation of the immune system, as did a bioprocessed mushroom formulation [229]?

### 8.3. Antiviral Activities

A pioneering study [230], discovered that nonsulphated and sulphated polysaccharides nearly completely inhibited cell-to-cell infection of human immunodeficiency viruses HIV-1 and HIV-2 and a human T-cell lymphotropic virus type 1 (HTLV-1). The following capsule summaries show that native and chemically modified mushroom polysaccharides exhibit antiviral activities against pathogenic viruses. A 120 kDa polysaccharide from the fruiting body of the *Pleurotus abalonus* mushrooms inhibited the HIV virus [157]. The melanin-glucan complex obtained from *Fomes fomentarius* mushrooms showed higher anti-HIV activity in comparison with the drug zidovudine in vitro and in rats and also exhibited antimicrobial properties against *Helicobacter pylori* (associated with human ulcers) with a greater efficacy than medicinal antibiotics [231]. The polysaccharide from *Agaricus brasiliensis* [232], and its sulphated derivative exhibited strong anti-herpes simplex virus activities [233,234]. A polysaccharide and extracts from the widely consumed *Lentinula edodes* (shiitake) mushrooms showed viricidal activity against both the bovine herpes simplex type 1 and poliovirus type 1 viruses [235]. Aqueous extracts from several mushroom varieties protected mice against lethality induced by the Herpes simplex type-2 virus [236], and a mushroom lentinan and its sulphated product protected tobacco seedlings against viral infection by the tobacco mosaic virus [237].

The cited observations indicate that mushroom polysaccharides and some of their chemically sulphated forms might protect against viral diseases that affect plants, animals, and humans.

## 9. Additional Health Benefits

Here we will briefly mention three additional health-promoting properties: protection against allergic asthma, adverse effect of γ-radiation, and enhancement of vitamin D content of mushroom by UV-B radiation.

### 9.1. Protection of Mice against Allergic Asthma

Because mushrooms can break down complex plant materials into smaller, more digestible and bioactive compounds, Kim, et al. [238] investigated the efficacy of a bioprocessed (fermented) liquid mycelium culture against allergic asthma in chicken egg ovalbumin-sensitized/challenged asthmatic mice. The observed inhibitory activity against ovalbumin-specific IgE secretion in bronchoalveolar lavage fluid and additional observations with several allergic asthma-associated biomarker levels suggested the production of new bioactive compounds by the mushroom mycelia that may be involved in enhancing the observed antiasthmatic properties. It seems that the elm tree (*Ulmus parvifolia*) bark bioprocessed with mycelia of shiitake (*Lentinus edodes*) mushrooms has the potential to prevent and/or treat allergic asthma. We do not know if mycelia polysaccharides contribute to the antiasthmatic properties of the bioprocessed formulation. In a related study, Kim, et al. [229] also discovered that turmeric bioprocessed with Shiitake mushroom mycelia protected mice against salmonellosis via stimulation of the immune system.

### 9.2. Protection of Mice against Adverse Effects of γ-Radiation

Because ionizing radiation damages cells by creating an imbalance in their prooxidant to antioxidant ratio, it was of interest to find out if antioxidative polysaccharides might protect against radiation-induced adverse effects. This seems to be the case as illustrated by the following observations: (a) Joseph, et al. [239] found that a polysaccharide-protein complex from *Phellinus rimosus* mushrooms administered intraperitoneally to mice exposed to γ-radiation resulted in enhanced levels of antioxidants in liver and brain tissues and in an increased survival of the animals; (b) A related study by Li, et al. [240] found that a polysaccharide from *Hohenbuehelia serotina* mushrooms induced an increase in the quantity of bone marrow, reduced the rates of chromosome aberrations and micronuclei in bone marrow, and blocked the apoptotic pathway of splenocytes in mice exposed to γ-radiation; and (c) A β-glucan from the mushroom *Ganoderma lucidum* extended the survival of mice exposed to a lethal dose of γ-radiation after 30 days by 66% post-irradiation compared to 83% observed with the anti-radiation drug amifostine [241].

These results with mice imply that the mentioned polysaccharides might protect humans against allergic asthma and radiation injuries. Will these and possibly other mushroom polysaccharides mitigate adverse side effects of radiation used to treat cancer patients [242]?

### 9.3. Effect of UV-B and on Vitamin D2 Content of Mushrooms

To facilitate industrial application of the known increase in vitamin D2 content by post-harvest exposure to ultraviolet B (UV-B) light, Roberts, et al. [243] carried out in this laboratory a detailed study of the effect of UV-B radiation on the formation and stability of the vitamin in *Agaricus bisporus* (Portabella) mushrooms. The following conditions were found to be optimal for practical commercial production of the mushrooms with a vitamin content of 3.75 µg/g: light intensity, 1.0 mW; dose, 0.5 J/cm; time of exposure, 8 min. The vitamin D2 content of the treated mushroom was, however, degraded during long-term storage with a rate constant of 0.025 h-1. Pulsed UV light (PUV) [244] and γ-radiation also increased the content of vitamin D2 in *Agaricus bisporus* mushrooms [245,246].

In related studies, (a) Sławińska, et al. [247] reported that after 1.5 year storage of dried *Agaricus bisporus* UV-B mushrooms, the vitamin D2 content was decreased by 48.32% of the initial level; (b) Mehrotra, et al. [248] describe unanticipated losses of D2 during cooking from fresh UV-B mushrooms; and (c) Calvo, et al. [249], Chen, et al. [250], and Simon, et al. [251] found that the vitamin D in mushrooms, produced by exposure to UV light, seemed to be bioavailable and safe.

A largely unanswered question is whether exposure of mushrooms to UV-B light might also affect different mushroom components, as illustrated by the following observation. A compositional study on the effect of UV-B radiation reports that exposure of the medicinal mushroom Cordyceps militaris to UV-B light for 2 hours resulted in an increase in vitamin D2 from 0–0.03 to 0.22–1.11 mg/g, a decrease in the vitamin D2 precursor ergosterol from 1.36 to 2.50 mg/g, an increase in the amount of adenosine, cordyceptin, and ergothioneine by 32-128% and in flavonoid content from 10 to 56%, and a 36% decrease in the polysaccharide levels of the fruiting bodies [252]. Whether UV-B radiation also adversely affects mushroom polysaccharides merits study.

## 10. Conclusions

The relationship between dietary content and disease is a major concern for human health. We anticipate that the findings described here on the beneficial health properties of individual mushroom polysaccharides and polysaccharide-containing mushrooms will contribute to improvements in nutrition, food safety, and human health. In view of an increased interest in the multiple bioactive components of mushrooms—for example, we reported that the *Hericium erinaceus* mushroom cultivars contain more than 50 characterized organic compounds many of which are bioactive [22]—there is a need to define further our current knowledge about the beneficial health-promoting properties and the active constituents, including the polysaccharides that have attracted widespread interest. In addition to research needs mentioned earlier, finding answers to unresolved aspects of mushroom polysaccharides is expected to benefit human health further. We are challenged to address the following research needs:

- Determine whether the antiobesity and antidiabetic properties of pure polysaccharides vary depending on whether they are tested or consumed in their free state or as part of a food.
- Determine the relationship between consumption of mushroom polysaccharides and lower risks of obesity and diabetes.
- Determine whether mushroom polysaccharide metabolites formed in the digestive tract and after absorption into the circulation possess antiobesity and antidiabetic properties.
- Determine whether the polysaccharide content of mushrooms can predict antiobesity and antidiabetic activities.
- Determine additive and synergistic antiobesity properties of combinations of mushroom polysaccharides and the antiobesity amino acid arginine [253,254].
- Define mitigating effects of conditions associated with the metabolic syndrome of combinations of mushroom polysaccharides with other bioactive natural products and compounds, including apples [255], bioactive potato and eggplant (aubergine) glycoalkaloids and potato calystegine alkaloids [256,257], tomatoes and tomato compounds [256,258,259,260,261,262], rice hull smoke extract [152,263,264,265], bioactive compounds from essential oils and spices [266], bioactive rice bran compounds [267], bioactive tea compounds [268,269], and red wine and winery byproducts [270,271,272].
- Determine bioactivities of mixtures of structurally different polysaccharides isolated from different mushroom species.
- Determine additive and synergistic health properties of combinations of dietary β-glucans derived from mushrooms and cereals.
- Determine additive and synergistic properties of mushroom polysaccharides in combination with antiobesity and antidiabetes drugs.
- Because mushrooms can break down complex plant materials into smaller compounds to produce additional bioactive compounds during bioprocessing (fermentation), determine if this novel approach can be extended to other combinations of mushrooms with polymeric materials present in tree barks and plant cell walls, a largely unexplored area of mushroom research [238].
- Explore the potential of dietary mushrooms to improve meat quality of poultry and beef products by decreasing lipid peroxidation [273,274].
- Because the preparation of low-sodium meat with improved flavor properties might help reduce hypertension [275], evaluate flavor-enhancing properties of mushrooms added to low-sodium meat-based food [146]. Develop flavor- and health- enhancing mushroom seasonings (powders) that can be added to food [276].
- Because diet-induced obesity in male mice and possibly also in male humans might be associated with reduced fertility caused by acrylamide-induced reproductive toxicity [277,278,279], determine if mushroom-induced reduction in human obesity will concurrently mitigate acrylamide-induced male infertility.
- Because growth hormone receptor deficiency in about 100 Ecuadorian adults seems to be associated with essentially no incidences of lifetime cancers and diabetes among these dwarfs, it might be worthwhile to find out the possible effect of polysaccharides on the growth hormone in the general population [280,281].
- Determine if mushroom polysaccharide-induced stimulation of the human immune system would help protect against susceptible and antibiotic-resistant foodborne (*E*. *coli*, *Listeria*, *Salmonella*) and medical (*Clostridium difficile*, *Klebsiella*, *Streptococci*) pathogenic bacteria and endotoxemia [31,92,93,282].
- Evaluate antibiotic properties of mushroom polysaccharides against food- and insect-borne viruses that cause human illness, including norovirus and Zika virus [283,284].
- Explore the inactivation of microbial and plant toxins by mushroom products [285,286], and
- Encourage researchers to create high-polysaccharide mushrooms using plant molecular biology methods and mushroom growers to produce mushrooms with a high content of polysaccharides.

In summary, because edible mushrooms and bioactive polysaccharides are considered to be generally accepted as safe, the cited and proposed studies could provide numerous benefits. Mushroom polysaccharides individually, added to food, and in combination with medicinal drugs could be used therapeutically to help prevent and protect against, at low cost, the adverse effects of some of the major diseases that afflict humans.

It would be interesting to find out whether the dietary polysaccharides and polysaccharide-containing mushroom extracts and powders might concurrently ameliorate multiple human disease syndromes associated with allergy, cancer, diabetes, infections, and obesity with common inflammatory and other underlying inflammatory, immune, and oxidative molecular and cellular biomarkers. Understanding of the overlapping chemical and pharmacological aspects of mushroom polysaccharides will provide valuable insights into their potential to prevent and treat chronic diseases as well as bacterial and viral infections. Moreover, because some mushroom polysaccharides might compensate for vitamin D and calcium deficiencies and the D vitamin of mushrooms can be increased by exposure to UVB light, dietary mushrooms can play a significant role in bone and cartilage health. The payoff would be the availability of inexpensive natural and safe functional foods and healthier humans. A next step is for physicians and other biomedical scientists to apply the available knowledge to help overcome such diseases.
